# The Role of Myofascial Release Techniques as an Adjunct to Other Therapies in Knee Osteoarthritis: A Systematic Review

**DOI:** 10.1002/hsr2.71507

**Published:** 2025-11-24

**Authors:** Farnaz Farshchi, Nader Farshchi, Reza Abdi Hamzeh Kalayi

**Affiliations:** ^1^ Department of Occupational Therapy, School of Rehabilitation Sciences Tehran University of Medical Sciences Tehran Iran; ^2^ Department of Physical Therapy, School of Rehabilitation Sciences Iran University of Medical Sciences Tehran Iran; ^3^ School of Medicine Tehran University of Medical Sciences Tehran Iran

**Keywords:** function, knee osteoarthritis, myofascial release, pain, range of motion

## Abstract

**Background and Aim:**

Myofascial dysfunctions have been recognized as a pain‐causing component in knee osteoarthritis (KOA). Recently, clinicians have focused on various therapeutic techniques targeting fascia. The myofascial release technique (MRT) aimed to address fascial restrictions and myofascial trigger points to improve range of motion (ROM) and muscle function before rehabilitation or physical activity. This systematic review aimed to evaluate the effectiveness and safety of MRT in individuals with KOA, to provide an evidence‐based foundation for their clinical application.

**Methods:**

PRISMA guidelines were performed for this review. Randomized or non‐randomized controlled trials were systematically collected from PubMed, Scopus, Web of Science, Science Direct, Google Scholar, and ProQuest from their inception until May 2025. The methodological quality of the articles was assessed using the PEDro checklist.

**Results:**

Of the 789 records identified, 12 were deemed relevant. The studies exhibited a mean quality score of 4–5 (acceptable) in six studies, a mean quality score of 3 (poor) in one study, and a mean quality score of 6–8 in five studies. The duration of MRT treatment ranged from 3 to 12 sessions, with most studies administering 12 sessions over a period of 2–6 weeks. The outcome measures included pain levels, knee flexion ROM, functional disability, muscle strength, quality of life, knee alignment, and dynamic knee valgus. Five trials compared MRT combined with conventional therapies to a conventional physiotherapy group alone, two trials compared MRT combined with conventional therapies to an MRT group alone, and five trials compared MRT to other therapeutic techniques.

**Conclusion:**

MRT, when used as an adjunct to conventional therapies or corrective exercises, was found to be more effective in reducing pain, enhancing functional ability, and improving knee range of motion in individuals with KOA. However, MRT alone did not demonstrate consistent superiority over other manual therapy techniques. Due to the concurrent use of multiple interventions in most studies, the specific contribution of MRT remains uncertain. Thus, MRT may be considered a potentially beneficial complementary therapy, but further high‐quality studies are needed to establish its standalone efficacy.

## 1 Introduction

Knee osteoarthritis (KOA) is the most commonly encountered condition affecting the musculoskeletal system. KOA is a leading cause of disability worldwide, affecting more than 22% of the global population aged over 40 years and approximately 250 million people overall [1]. Its prevalence increases substantially with age, obesity, and female gender, making it a growing health concern in both developed and developing countries [2]. This condition is a complex disorder with multiple causes, varying clinical presentations, and a degenerative pathology that also exhibits some regenerative features, including chondrocyte proliferation, subchondral bone remodeling, and osteophyte formation [3]. Its signs and symptoms can change over time, resulting in different possible outcomes for the disease process [4].

A trigger point is a sensitive area in the muscle or connective tissue (fascia) that becomes painful when pressure is applied. Pressing on a trigger point can lead to referred pain and assist in identifying the external area of the body that is generating the pain [5]. Various studies indicate that pain and limitations are primarily caused by myofascial trigger points in the muscles [6, 7], which can be treated through myofascial release therapy [8, 9]. This treatment may help prevent disability. In KOA, pain and dysfunction can arise not only from these myofascial abnormalities but also from the underlying degenerative changes in the articular cartilage, subchondral bone, and periarticular structures. Such degeneration alters joint biomechanics and loading patterns, leading to abnormal tension, overuse, and ischemia in periarticular and thigh muscles, particularly the quadriceps, hamstrings, and iliotibial band [10]. These conditions favor the development of myofascial trigger points, which in turn exacerbate pain, reduce flexibility, and contribute to functional limitations, reinforcing the cycle between joint degeneration and myofascial dysfunction [4].

Myofascial release is a passive stretching technique that targets the fascia, helping to release tension in the superficial layer between the muscles and the fascia. This technique is a form of manipulation that promotes stretching and increases the extensibility of the tissue [11, 12, 13]. Among the various methods that work on fascial tissue structures, myofascial release technique (MRT) has been recognized for its potential to reduce pain, improve flexibility, decrease disability, and enhance overall function in daily activities [14]. MRT is thought to modulate pain perception through mechanisms such as pressure‐induced gate control, relief of muscle spasms, enhanced blood circulation, and improved tissue extensibility [15, 16]. Emerging evidence also highlights the role of MRT in improving flexibility, neuromuscular performance, and fascial mobility in active and clinical populations [17, 18], providing further rationale for its application in musculoskeletal conditions such as KOA [19]. These physiological effects support its growing use as a complementary approach in musculoskeletal rehabilitation. Numerous systematic reviews have confirmed the effectiveness of MRTs in managing various musculoskeletal disabilities such as chronic low back pain, fibromyalgia, and neck pain by reducing pain, improving muscle function, enhancing performance, and promoting recovery [20, 21, 22, 23, 24]. Although individual studies have investigated the application of MRT in patients with KOA, to our knowledge, no prior systematic review has synthesized these findings. Given this high disease burden, there is a growing need for conservative interventions that can effectively reduce pain and disability. The MFR, by addressing fascial restrictions and myofascial trigger points, may improve flexibility, reduce pain, and enhance joint function in KOA. These potential benefits justify the investigation of MFR as an adjunctive therapeutic approach [9, 19, 25]. This systematic review aimed to evaluate the effectiveness and safety of MRT in individuals with KOA using an evidence‐based approach, thereby providing a scientific foundation for its clinical application in this context.

## 2 Methods

### 2.1 Registration of Protocol

This systematic review was conducted following the Preferred Reporting Items for Systematic Reviews and Meta‐Analyses (PRISMA) checklist (Supporting Information: Appendix 1). The protocol for the systematic review was preregistered in PROSPERO before data extraction began (CRD420251044596).

### 2.2 Search Strategy and Selection of Studies

The literature search was conducted according to the participants, intervention, comparators, and outcomes (PICO) criteria by two independent researchers, F.F. and R.A.H.K. Any discrepancies between the researchers were resolved through discussions with a third researcher, N.F. We searched several electronic databases, including the Science Direct, PubMed, Scopus, ProQuest, Google Scholar, and Web of Science, to identify relevant articles published from their inception until May 2025. The search strategy for each database has been presented in Supporting Information: Appendix 2. Ultimately, F.F. and R.A.H.K. independently identified the studies that met the inclusion and exclusion criteria.

### 2.3 Eligibility Criteria

All studies meeting the following criteria were included in this study: (i) Participants: patients diagnosed with KOA; (ii) Intervention: the experimental group received MRT; (iii) Control: studies included active or passive control groups compared to MRT, with no limitations on the intervention for the control group; and (iv) Study type: only clinical randomized controlled trials or quasi‐experimental trials. No language restrictions were applied during the search process. Exclusion criteria included duplicate articles, studies with incomplete data, case reports, review articles, and animal experiments.

### 2.4 Risk of Bias and Methodological Quality Assessment

To assess the quality of the studies, we utilized the Physiotherapy Evidence Database (PEDro) tool [26], primarily based on the independent consensus of the authors F.F. and R.A.H.K. If there was any scoring discrepancy between these two independent researchers, a third researcher, N.F., intervened to resolve the disagreement. The PEDro scale consists of 11 criteria, with one point awarded for each clearly met criterion. While the eligibility criterion is not included in the score, it is part of the scale for evaluating external validity. According to a prior article, a study with a PEDro score of 6 or higher is classified as level 1 evidence (scores of 6–8 = good, 9–10 = excellent). Conversely, a study with a score of 5 or lower is categorized as level 2 evidence (scores of 4–5 = acceptable, and scores below 4 = poor) for the purposes of the present study [27] (Table 1).

**Table 1:** Quality assessment.

	Authors (year)	Q1. Eligibility criteria	Q2. Subjects' randomization into groups	Q3. Concealing allocation	Q4. Groups were homogenous at baseline regarding the most important prognostic factors	Q5. Subjects' blinding	Q6. Therapists' blinding	Q7. Assessors' blinding	Q8. At least, one main outcome was measured from more than 85% of the individuals initially allocated to groups	Q9. All subjects for whom outcome measures were available received the treatment or control condition as allocated. Data for at least one main outcome was analyzed by “intention to treat”	Q10. Findings of between‐group statistical comparisons were presented for at least one main outcome	Q11. Providing both point measurements and variability measurements for at least one main outcome	Total score	Quality status
1	Sharmin (2023) [28]	Yes	Yes	No	No	Yes	No	No	Yes	Yes	Yes	No	5	Acceptable
2	Rahman (2023) [29]	Yes	Yes	No	Yes	Yes	No	No	Yes	Yes	Yes	No	6	Good
3	Rahman (2023) [30]	Yes	Yes	No	Yes	No	No	Yes	Yes	Yes	Yes	No	6	Good
4	Nouman (2024) [19]	Yes	Yes	No	Yes	No	No	Yes	Yes	Yes	Yes	No	6	Good
5	Kusumawati (2024) [31]	Yes	Yes	No	Yes	No	No	No	Yes	Yes	Yes	No	5	Acceptable
6	Rahbar (2012) [25]	Yes	Yes	No	Yes	No	No	Yes	Yes	Yes	Yes	No	6	Good
7	Sonali (2022) [32]	Yes	No	No	No	Yes	No	No	Yes	Yes	Yes	No	4	Acceptable
8	Dixit (2020) [33]	Yes	Yes	No	No	No	No	No	Yes	Yes	Yes	No	4	Acceptable
9	Wafiq (2020) [34]	Yes	No	No	No	No	No	No	Yes	Yes	Yes	No	3	poor
10	Punjani (2025) [35]	Yes	Yes	No	No	No	No	Yes	Yes	Yes	Yes	No	5	Acceptable
11	Hamed (2024) [36]	Yes	Yes	No	Yes	Yes	No	Yes	Yes	Yes	Yes	No	7	Good
12	Mahmooda (2020) [37]	Yes	Yes	No	Yes	No	No	No	Yes	Yes	Yes	No	5	Acceptable

### 2.5 Data Extraction and Synthesis

Data extraction was performed independently by two reviewers (F.F. and R.A.H.K.). Any discrepancies were discussed and resolved through consultation with a third reviewer (N.F.). Extracted data were organized and stored in Microsoft Excel spreadsheets to ensure consistency and traceability. The following data were extracted from each selected study: authors' names, study design, participant characteristics, duration of the MRT intervention, regions treated with the MRT technique, outcome measures, and conclusions. The significance level (*p* value) for all studies was considered to be *p* < 0.05 based on the thresholds used in the original studies included in this review. In addition, information on whether studies reported a priori power calculations and their primary outcomes was extracted whenever available. Data synthesis followed PRISMA guidelines, and results were summarized narratively.

### 2.6 Search Summary

**Figure 1:**
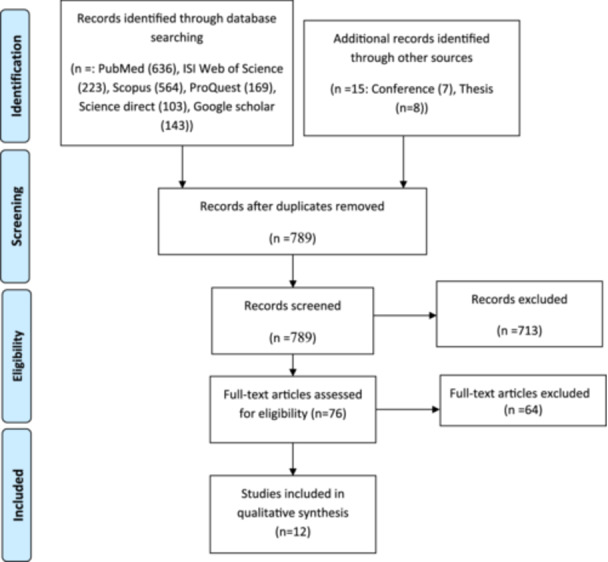
Flow diagram of the study selection based on PRISMA.

A flowchart outlining the selection of studies according to PRISMA guidelines is presented in Figure 1. Initially, 1853 articles were identified from the databases. After eliminating duplicate entries by endnote software, 789 articles remained for screening. Based on the screening of titles and abstracts, 76 articles were selected for full‐text review. Subsequently, 64 articles were excluded because they did not meet the inclusion/exclusion criteria. Ultimately, 12 studies were included in this systematic review. Among the included studies, 11 were published in English and one [25] in Persian.

## 3 Results

### 3.1 Characteristics of Studies

In the 12 studies that met the inclusion criteria for this review, a total of 445 individuals with KOA were analyzed, comprising 128 men and 245 women, with ages ranging from 35 to 75 years. The study did not specify the percentage of women and men with KOA in one instance [33]. The studies had a mean quality score of 4–5 (acceptable) [28, 31, 32, 33, 35, 37], a score of 3 (poor) [34], and a score of 6–8 (good) [19, 25, 29, 30, 36] on the PEDro scale, which is out of 10 (Table 1).

Regarding study design, nine studies were randomized controlled trials, while two were non‐randomized clinical trials or quasi‐experimental studies (Table 2). Among the 12 studies, five implemented assessor blinding [19, 25, 30, 35, 36], and four employed patient blinding [28, 29, 32, 36]. The remaining studies did not include any form of blinding [31, 33, 34].

**Table 2:** Characteristics of included studies.

Author (year)	Study design	Participants	Intervention	Intervention duration of MRT	Treated regions of MRT technique	Outcome measures	Conclusion
Sharmin (2023) [28]	– Single blind (patient) RCT	– 22 individuals (13 males, 9 females) with KOA, age: 43–70 years, grade II or III – Control group (*n* = 11) – Experimental group (*n* = 11)	– Control group: Only conventional physiotherapy – ‐Experimental group: MRT with conventional physiotherapy	12 sessions, 5 min per session with 3–4 days per week	Vastus medialis, vastus lateralis, iliotibial band, gastrocnemius	– Pain (NPRS) – ‐ROM (goniometer) – Muscle strength (MMT) – Functional disability (WOMAC)	MRT combined with conventional physiotherapy was more effective than conventional physiotherapy alone on the outcome measured for patients with KOA
Rahman (2023) [29]	– Single blind (patient) RCT	– 32 individuals (15 males, 17 females) with KOA, age: 35–60 years, grade II or III – Control group (*n* = 16) – Experimental group (*n* = 16)	– Control group: Band band MRT and baseline treatment only – Experimental group: Iliotibial Band band MRT other than the stretching and baseline treatment	12 sessions, 30 min	Iliotibial band	– Pain (VAS) – Functional disability (WOMAC)	The iliotibial band MRT along with stretching had statistically significant effect than control group in improving the pain and disability among KOA patients
Rahman (2023) [30]	– Single blind (assessor) RCT	– 0 individuals (23 males, 7 females) with KOA, age: 50–70 years, grade I, II, III, IV– Group A (*n* = 15) – Group B (*n* = 15)	– Group A: Physio gun release with usual physiotherapy – Group B: MRT with usual physiotherapy	12 sessions, 3 days per weeks over 4 weeks, 3–5 min per session for each muscle	Iliotibial band, vastus medialis, vastus lateralis	– Pain (VAS) – ‐ROM (goniometer) – Functional disability (WOMAC)	Physio gun release along with usual physiotherapy were more effective therapeutic approach for patients with knee OA
Nouman (2024) [19]	– Single blind (assessor) RCT	– Control group (*n* = 18) – Experimental group (*n* = 18)	– Control group: MRT alone – Experimental group: MRT with valgus correction exercises	12 sessions, 40 min per session, twice weekly over 6 weeks	Iliotibial band	– Pain (VAS) – ROM (goniometer) – Balance (BBS) – Quality of life (KOOS)	MRT combined with valgus correction exercises compared with MRT alone can effectively improve the pain, ROM, balance, and quality of life in patients with KOA
Kusumawati (2024) [31]	– No blinding RCT	– 30 individuals (6 males, 24 females) with KOA, age: 40–60 years, grade I – Control group (*n* = 15) – Experimental group (*n* = 15)	– Control group: Standard therapy – Experimental group: Standard therapy along with MRT	12 sessions over 4 weeks	Iliotibial band	– Pain (VAS) – Functional disability (WOMAC)	There is a significant difference in effect between standard therapy and standard therapy plus MRT of the iliotibial band to improve pain and to increase functional activity in KOA
Rahbar (2012) [25]	– Single blind (assessor) RCT	– 60 individuals (11 males, 49 females) with KOA, age: over 50 years, grade: NS – Control group (*n* = 30) – Experimental group (*n* = 30)	– Control group: Standard therapy – Experimental group: Standard therapy along with MRT	16 sessions	Quadriceps, hip adductors, iliotibial band, tensor fascia lata, hamstrings, and calf muscles	– Pain (VAS) – ROM (goniometer) – Functional disability (WOMAC) – Physical efficiency (TUG)	Comparison of the two groups showed that, except for ROM, the experimental group was better than the control group in all outcome measures
Sonali (2022) [32]	– Single blind (patient) quasi experimental	– 30 individuals (17 males, 13 females) with KOA, age: 40–75 years, grade: NS – Control group (*n* = 15) – Experimental group (*n* = 15)	– Control group: Standard therapy – Experimental group: Standard therapy along with MRT	12 sessions over 4 weeks, 3days per week	Quadriceps (vastus medialis, vastus lateralis, rectus femoris, and vastus Intermedius)	– Pain (VAS) – Functional disability (WOMAC)	Both methods of conventional physiotherapy combined with quadriceps MRT and conventional physiotherapy alone were successful in improving pain and disability of KOA
Dixit (2020)[33]	No‐blinding RCT	– 72 individuals (male: NS, female: NS) with KOA, age: 40–60 years, grade: I and II – Group A (*n* = 36) – Group B (*n* = 36)	– Group A: Maitland mobilization with conventional therapy – Group B: MRT with conventional therapy	3 days alternately a week for 6 weeks for 30–35 min	Iliotibial band, quadriceps, and hamstrings	– Pain (VAS) – ROM (goniometer) – Functional disability (WOMAC)	Maitland mobilization and conventional therapy were more effective than MRT in relieving pain, improving ROM, and functional disability in subjects with KOA
Wafiq (2020) [34]	Quasi‐experimental design with a nonequivalent group approach	– 25 individuals (2 males: 23 females) with KOA, age: 47–79 years, grade: NS – Control group (*n* = 14) – Experimental group (*n* = 11)	– Control group: Kinesio Taping – Experimental group: Combination of MRT and Kinesio taping	3 sessions a week in one month	NS	– ROM (goniometer)	The results showed no significant difference in the effect of the treatment of Kinesio taping and the combination of MRT and Kinesio taping alone in increasing ROM in the elderly with KOA
Punjani (2025) [35]	– Single blind (assessor) RCT	– 32 individuals (13 males: 19 females) with KOA, age: 40–60 years, grade: II and III – Group A (*n* = 16) – Group B (*n* = 16)	– Group A: Deep front line MRT with conventional therapy – Group B: Kinetic chain activation technique with conventional therapy	6 session over 2 weeks	Quadratus lumborum, abdominal fascia, and diaphragm, iliacus, and psoas major	– Pain (NPRS) – Dynamic knee valgus – Knee (videographic analysis) – Knee alignment (x‐ray) – Quality of life (KOOS)	Both deep front line MRT and kinetic chain activation technique were found to be equally effective in alleviating pain, improving quality of life, and knee malalignments
Hamed (2024) [36]	Double blind (patient and assessor) RCT	– 42 individuals (13 males: 29 females) with KOA, age: 45–60 years, grade: II and III – Control group (*n* = 21) – Experimental group (*n* = 21)	– Control group: Exercises of stretching – Experimental group: Deep line MRT	12 session, 3 times per week over 4 weeks	Tibialis posterior, hip adductors	– Pain (VAS) – ROM (goniometer) – Functional disability (WOMAC)	In patients with KOA, adding deep line MRT to stretching exercises resulted in superior outcomes compared to using stretching exercises alone
Mahmooda (2020) [37]	No‐blinding RCT	– 30 female individuals with KOA, age: 40–60 years, grade: II – Group A (*n* = 15) – Group B (*n* = 15)	– Group A: Mulligan's mobilization with movements along with conventional exercise – Group B: MRT along with conventional exercise	10 sessions, 5 days a week, once a day over 2 weeks	Hamstrings, quadriceps	– Pain (NPRS) – ROM (goniometer) – Functional disability (WOMAC)	Both Mulligan's mobilization with movement and MRT were effective in managing pain in individuals with KOA. However, Mulligan's therapy proved to be more effective than MRT in reducing pain and improving ROM. Conversely, MRT was found to be more effective than Mulligan's therapy in addressing stiffness and enhancing functional abilities

Abbreviations: BBS, Berg balance scale; KOOS, knee injury and osteoarthritis outcome score; MMT, Manual muscle testing; MRT, myofascial release technique; NPRS, numeric pain rating scale; NS, not stated; RCT, randomized controlled trials; ROM, range of motion; TUG, timed up and go; VAS, visual analogue scale; WOMAC, Western Ontario and McMaster Universities arthritis index.

The treatment duration for MRT varied across the studies, ranging from 3 to 12 sessions, with most studies comprising 12 sessions completed over 2–6 weeks. Generally, the MRT targeted various muscles, including the quadriceps (vastus medialis, vastus lateralis, and vastus intermedius), iliotibial band, gastrocnemius, hip adductors, tensor fascia lata, hamstrings, calf muscles, quadratus lumborum, abdominal fascia, diaphragm, iliacus, psoas major, and tibialis posterior. In most included studies, MRT was applied alongside conventional therapies such as physiotherapy or corrective exercises. However, in a few studies, one group received MRT without adjunctive interventions [19, 29], allowing for limited comparisons between MRT alone and MRT combined with other therapies. None of the included studies reported any adverse events related to MRT in individuals with KOA.

Among the included studies, only two trials explicitly reported an a priori sample size calculation. Nouman et al. calculated the required sample size using the Giga calculator based on 80% power, a 5% confidence interval, and a 5% margin of error, but did not specify the primary outcome on which the calculation was based [1]. Similarly, Rahman et al. performed a power analysis using a 5.78% prevalence rate of knee OA in Bangladesh, with *α* = 0.05% and 80% power, though without reporting the specific outcome variable used for powering the study [30]. None of the other included studies provided details on power calculations or primary outcomes.

### 3.2 Outcomes Measures

The outcome measures included pain levels, knee flexion range of motion (ROM), functional disability, muscle strength, quality of life, knee alignment, and dynamic knee valgus. Pain was assessed using the Visual Analogue Scale (VAS) or the Numeric Pain Rating Scale (NPRS). Functional disability was evaluated with the Western Ontario and McMaster Universities Arthritis Index (WOMAC). ROM was measured using a goniometer. Quality of life was assessed with the Knee Injury and Osteoarthritis Outcome Score (KOOS). Knee alignment was measured using videographic analysis or X‐ray imaging. Muscle strength was evaluated through manual muscle testing (MMT), and physical efficiency was assessed using the Timed Up and Go (TUG) test.

### 3.3 Effects of MRT on Clinical Outcomes

#### 3.3.1 MRT Along With Conventional Therapies Compared With Conventional Therapies Alone

Five studies (quality: acceptable to good) compared the effectiveness of MRT combined with conventional therapies to that of a conventional physiotherapy group alone [25, 28, 31, 32, 36]. Among these, three studies (quality: acceptable to good) found that MRT in conjunction with conventional physiotherapy was more effective than conventional physiotherapy alone in improving pain, ROM, functional disability, and muscle strength in patients with KOA [28, 31, 36]. Another study (quality: good) indicated that, except for knee ROM, the experimental group receiving MRT alongside standard therapy demonstrated better results than the control group that received only standard therapy in terms of pain relief, functional disability, and physical efficiency [25]. However, Sonali et al. (quality: acceptable) reported that both conventional physiotherapy combined with quadriceps MRT and conventional physiotherapy alone are effective in reducing pain and disability in patients with KOA, with neither treatment showing greater superiority over the other [32].

#### 3.3.2 MRT Along With Conventional Therapies Compared With MRT Alone

Two studies (quality: good) compared MRT in conjunction with conventional therapies to MRT alone [19, 29]. One study examined the effects of iliotibial band MRT, with and without stretching, on patients experiencing iliotibial band tightness due to KOA. The results indicated that iliotibial band MRT combined with stretching significantly improved pain and disability compared to MRT alone [29]. Additionally, another study found that MRT, together with valgus correction exercises, effectively enhanced pain levels, ROM, balance, and overall quality of life for participants with KOA [19].

#### 3.3.3 Comparison of MRT With Other Therapeutic Techniques

When comparing MRT to other interventions, one study (quality: good) found that the use of a physio gun alongside usual physiotherapy proved to be more effective than MRT for reducing pain, improving ROM, and enhancing functional ability in patients with KOA [30]. Another study (quality: acceptable) evaluated Mulligan's mobilization with movement against MRT and reported that Mulligan's therapy was more effective than MRT in reducing pain and improving ROM. However, MRT demonstrated superior effectiveness compared to Mulligan's therapy when it came to stiffness and functional abilities [37]. A separate study (quality: acceptable) assessed the Maitland mobilization technique in comparison to MRT and concluded that Mulligan's therapy was significantly more effective than MRT in terms of pain relief, ROM, and functional disability [33]. In contrast, a study (quality: poor) examined the effects of a combination of MRT and Kinesio taping against Kinesio taping alone, finding no significant difference between the two interventions in increasing ROM among the elderly at risk of KOA [34]. Additionally, one study (quality: acceptable) compared the deep front line MRT and kinetic chain activation technique, reporting that both techniques, when used in conjunction with conventional therapy, were equally effective in alleviating pain, improving quality of life, and addressing knee malalignments [35].

### 3.4 Synthesis of Evidence

Overall, the findings of the included studies suggest that MRT may be beneficial as an adjunctive therapy in patients with KOA. Across most trials, MRT combined with conventional therapies demonstrated greater improvements in pain, ROM, and functional ability compared to conventional therapies alone. However, when MRT was applied in isolation, the results were less consistent, and its superiority over other manual therapy techniques (e.g., Mulligan or Maitland mobilization) was not clearly established. Regarding risk of bias, as assessed by the PEDro scale, one trial was rated poor, six were acceptable, and five were good (Table 1). Thus, the overall body of evidence can be considered of low‐to‐moderate risk of bias, with most studies providing acceptable to good methodological quality.

## 4 Discussion

Given the widespread use of MRT methods in treating orthopedic diseases, this is the first systematic review aimed at evaluating the effectiveness and safety of MRT in individuals with KOA. While several studies reported improvements in pain, ROM, and functional disability following interventions that included MRT, most applied MRT in conjunction with other therapies. Therefore, it remains unclear whether the observed benefits can be attributed solely to MRT, to the accompanying treatments, or to a synergistic effect of both. MRT may be considered a potentially effective adjunctive therapy; however, current evidence is limited in determining its isolated effects due to the methodological designs of the included studies.

The trials included in this review showed that participants who received MRT in combination with corrective exercises achieved more significant improvements compared to those who received corrective exercises alone [28, 31, 36] or MRT without adjunctive therapies [19, 29]. While MRT was not evaluated in complete isolation in any study, some comparisons involved groups receiving MRT without any added therapeutic modalities, thereby offering partial insight into its individual contribution. KOA is commonly associated with pain, limited ROM, and reduced joint function. Among various conservative treatment options, exercise therapy remains a core component, commonly used in combination with other interventions such as joint mobilization or electrotherapeutic modalities [38]. In most of the included studies, the conventional therapy groups received multi‐modal treatments, often with exercise as the primary or foundational element. A study by Sonali et al. reported that the experimental group, which received quadriceps MRT combined with conventional physiotherapy, experienced a greater reduction in pain and disability compared to those receiving conventional physiotherapy alone; however, this difference was not statistically significant [32]. One possible reason for this could be that Sonali's study focused solely on the quadriceps muscle [32], neglecting other important muscles such as the iliotibial band, hamstrings, hip abductors, and calf muscles. Based on these findings, we conclude that MRT, when combined with traditional physiotherapy, may be effective in alleviating symptoms in patients with KOA, thereby aiding their rehabilitation and improving their functional abilities.

When comparing MRT with Mulligan's therapy [37], Maitland mobilization [33], or physio gun release [30], studies have indicated that each of these interventions leads to quicker improvements in pain and ROM than MRT. Manual therapy is commonly used as a physical treatment for KOA. Both Maitland and Mulligan mobilization techniques are two types of manual therapy applied in the treatment of KOA [39, 40]. Mulligan mobilization allows patients to perform the movements that cause discomfort while maintaining a functional position, which can lead to positive outcomes [41]. In contrast, Maitland mobilization focuses on restoring the proper spinning, gliding, and rolling motions between the joints [40]. A systematic review and meta‐analysis of the effectiveness of Maitland and Mulligan mobilization methods for adults with KOA demonstrated that the Mulligan mobilization technique is a promising intervention for alleviating pain and improving functional scores in KOA patients [39]. On the other hand, physio gun release is a technique that involves using a device to provide massage directly to the muscles, which helps relieve pain and increase ROM [42]. Studies have shown that vibrating foam rollers can significantly impact muscle soreness, while vibration therapy has been effective in reducing pain, enhancing pain thresholds, and improving range of motion [43]. The physio gun is a device similar to a vibrating foam roller [30]. In MRT, a sustained deep frictional stretch is applied along the muscles. It is worth noting that the study by Wafiq et al. [34], which examined the combined use of MRT and Kinesio taping versus Kinesio taping alone, received a quality rating of 3 (poor) in our assessment. Although it offered a unique perspective by evaluating a novel combination of interventions, its methodological limitations suggest that its findings should be interpreted with caution. However, since the effects of Mulligan's therapy, Maitland mobilization, physio gun release, or Kinesio taping in comparison to MRT have only been examined in a single study, more data and monitoring will be necessary to accurately assess their efficacy in real‐world and epidemiological contexts.

## 5 Limitations and Suggestions

This review has several limitations. First, the included studies showed considerable variability in terms of population characteristics, intervention protocols, and outcome measures. While our broad inclusion criteria were intended to capture the diversity of MRT applications in KOA, this approach also contributed to clinical heterogeneity that may have limited direct comparisons across studies. In hindsight, a more narrowly defined set of inclusion criteria may have helped reduce such variability. Second, some studies combined MRT with other interventions, making it difficult to isolate the specific effects of MRT. Third, none of the included studies clearly specified the primary outcomes for which they were powered, and most did not provide any information on statistical power. This raises concerns about the adequacy of sample sizes and increases the risk of type II error, thereby limiting the strength of the available evidence. Fourth, since none of the included trials reported on adverse events, the safety profile of MRT in KOA remains unclear.

Future studies should aim to investigate the long‐term effects and carryover benefits of MRT, especially considering that KOA is a progressive and non‐curable condition. Additionally, MRI could be utilized to track the movement of the patella during dynamic actions. Finally, studies that combine MRT with other treatment modalities are essential for a comprehensive understanding of its effects.

## 6 Conclusion

This systematic review suggests that MRT, when applied alongside conventional physiotherapy or corrective exercises, may be associated with improvements in pain, ROM, and functional ability in individuals with KOA. However, due to the concurrent use of other interventions in most studies, the independent effect of MRT remains unclear. Additionally, when MRT was compared with other manual therapy techniques, the results were mixed, with no consistent evidence of superiority. Therefore, MRT should be considered a potentially useful adjunctive therapy, though further high‐quality research is needed to clarify its specific contribution.

## Author Contributions

**Farnaz Farshchi:** conceptualization, investigation, methodology, writing – review and editing, writing – original draft. **Nader Farshchi:** methodology, writing – original draft, conceptualization. **Reza Abdi Hamzeh Kalayi:** investigation, project administration, supervision, writing – review and editing.

## Conflicts of Interest

The authors declare no conflicts of interest.

## Transparency Statement

The lead author, Farnaz Farshchi, affirms that this manuscript is an honest, accurate, and transparent account of the study being reported; that no important aspects of the study have been omitted; and that any discrepancies from the study as planned (and, if relevant, registered) have been explained.

## Supporting information

Supplemental appendix.

Supplemental appendix.

## Data Availability

Data sharing not applicable to this article as no data sets were generated or analysed during the current study.
