# Supplementary material for: The Role of Myofascial Release Techniques as an Adjunct to Other Therapies in Knee Osteoarthritis: A Systematic Review
Source: Health Sci Rep. 2025 Nov 24;8(11):e71507. doi: 10.1002/hsr2.71507 (PMC12641147; doi:10.1002/hsr2.71507)
Supplement: Supplementary file 1 — Supplemental appendix. [file HSR2-8-e71507-s001.docx]

**PubMed**

((((((Knee Osteoarthritis) OR (Osteoarthritis of the knee)) OR (Knee Osteoarthritides)) OR (Osteoarthritis of Knee)) OR (Osteoarthritis of Knees)) OR (Knee Arthritis)) AND ((((((((myofascial release) OR (muscle manipulation)) OR (myofascial therapy)) OR (fascial manipulation)) OR (trigger point therapy)) OR (deep tissue massage)) OR (active release technique)) OR (myofascial treatment))

**Google scholar**

(“Knee Osteoarthritis” OR “Osteoarthritis of the knee” OR “Knee Osteoarthritides” OR “Osteoarthritis of Knee” “Osteoarthritis of Knees” OR “Knee Arthritis”) AND (“myofascial release” OR “muscle manipulation” OR “myofascial therapy” OR “fascial manipulation” OR “trigger point therapy” OR “deep tissue massage” OR “active release technique” OR “myofascial treatment”)

**Science direct**

(“Knee Osteoarthritis” OR “Osteoarthritis of the knee” OR “Osteoarthritis of Knee” OR “Osteoarthritis of Knees”) AND (“myofascial release” OR “muscle manipulation” OR “myofascial therapy” OR “fascial manipulation” OR “trigger point therapy”)

**Scopus**

(ALL(“Knee Osteoarthritis”) OR ALL(“Osteoarthritis of the knee”) OR ALL(“Knee Osteoarthritides”) OR ALL(“Osteoarthritis of Knee”) OR ALL(“Osteoarthritis of Knees”) OR ALL(“Knee Arthritis”)) AND (ALL(“myofascial release”) OR ALL(“muscle manipulation”) OR ALL(“myofascial therapy”) OR ALL(“fascial manipulation”) OR ALL(“trigger point therapy”) OR ALL(“deep tissue massage”) OR ALL(“active release technique”) OR ALL(“myofascial treatment”))

**Web of science**

(((((ALL=(Knee Osteoarthritis)) OR ALL=(Osteoarthritis of the knee)) OR ALL=(Knee Osteoarthritides)) OR ALL=(Osteoarthritis of Knee)) OR ALL=(Osteoarthritis of Knees)) OR ALL=(Knee Arthritis) AND (((((((ALL=(myofascial release)) OR ALL=(muscle manipulation)) OR ALL=(myofascial therapy)) OR ALL=(fascial manipulation)) OR ALL=(trigger point therapy)) OR ALL=(deep tissue massage)) OR ALL=(active release technique)) OR ALL=(myofascial treatment)

**ProQuest**

(“Knee Osteoarthritis” OR “Osteoarthritis of the knee” OR “Knee Osteoarthritides” OR “Osteoarthritis of Knee” “Osteoarthritis of Knees” OR “Knee Arthritis”) AND (“myofascial release” OR “muscle manipulation” OR “myofascial therapy” OR “fascial manipulation” OR “trigger point therapy” OR “deep tissue massage” OR “active release technique” OR “myofascial treatment”)
