# Supplementary material for: The Role of Myofascial Release Techniques as an Adjunct to Other Therapies in Knee Osteoarthritis: A Systematic Review
Source: Health Sci Rep. 2025 Nov 24;8(11):e71507. doi: 10.1002/hsr2.71507 (PMC12641147; doi:10.1002/hsr2.71507)
Supplement: Supplementary file 2 — Supplemental appendix. [file HSR2-8-e71507-s002.docx]

**Supplemental Appendix 1: PRISMA checklist**

| **Section and Topic** | **Item #** | **Checklist item** | **Location where item is reported** |
| --- | --- | --- | --- |
| **TITLE** | | |  |
| Title | 1 | Identify the report as a systematic review. | Page 2 |
| **ABSTRACT** | | |  |
| Abstract | 2 | See the PRISMA 2020 for Abstract checklist. | Page 2 |
| **INTRODUCTION** | | |  |
| Rationale | 3 | Describe the rationale for the review in the context of existing knowledge. | Pages 3 and 4, the third and fourth paragraph of introduction |
| Objectives | 4 | Provide an explicit statement of the objective(s) or question(s) the review addresses. | Page 4, the last paragraph of introduction |
| **METHODS** | | |  |
| Eligibility criteria | 5 | Specify the inclusion and exclusion criteria for the review and how studies were grouped for the syntheses. | Page 5 (2.3. Eligibility Criteria) |
| Information sources | 6 | Specify all databases, registers, websites, organisations, reference lists and other sources searched or consulted to identify studies. Specify the date when each source was last searched or consulted. | Page 4 (2.1. Registration of Protocol) |
| Search strategy | 7 | Present the full search strategies for all databases, registers and websites, including any filters and limits used. | Supplemental Appendix 2 |
| Selection process | 8 | Specify the methods used to decide whether a study met the inclusion criteria of the review, including how many reviewers screened each record and each report retrieved, whether they worked independently, and if applicable, details of automation tools used in the process. | Pages 4 and 5 |
| Data collection process | 9 | Specify the methods used to collect data from reports, including how many reviewers collected data from each report, whether they worked independently, any processes for obtaining or confirming data from study investigators, and if applicable, details of automation tools used in the process. | Pages 6 and 7, data extraction |
| Data items | 10a | List and define all outcomes for which data were sought. Specify whether all results that were compatible with each outcome domain in each study were sought (e.g. for all measures, time points, analyses), and if not, the methods used to decide which results to collect. | Page 8 (3.2. Outcomes Measures) |
|  | 10b | List and define all other variables for which data were sought (e.g. participant and intervention characteristics, funding sources). Describe any assumptions made about any missing or unclear information. | Page 8 (3.2. Outcomes Measures) |
| Study risk of bias assessment | 11 | Specify the methods used to assess risk of bias in the included studies, including details of the tool(s) used, how many reviewers assessed each study and whether they worked independently, and if applicable, details of automation tools used in the process. | Page 5, (2.4. Assessment of the Methodological Quality of the Studies) |
| Effect measures | 12 | Specify for each outcome the effect measure(s) (e.g. risk ratio, mean difference) used in the synthesis or presentation of results. | Not applicable |
| Synthesis methods | 13a | Describe the processes used to decide which studies were eligible for each synthesis (e.g. tabulating the study intervention characteristics and comparing against the planned groups for each synthesis (item #5)). | Figure 1 and page 5 (2.7. Search Summary) |
|  | 13b | Describe any methods required to prepare the data for presentation or synthesis, such as handling of missing summary statistics, or data conversions. | Not applicable |
|  | 13c | Describe any methods used to tabulate or visually display results of individual studies and syntheses. | Table 2 |
|  | 13d | Describe any methods used to synthesize results and provide a rationale for the choice(s). If meta-analysis was performed, describe the model(s), method(s) to identify the presence and extent of statistical heterogeneity, and software package(s) used. | NO(meta-analysis was not applicable) |
|  | 13e | Describe any methods used to explore possible causes of heterogeneity among study results (e.g. subgroup analysis, meta-regression). | NO(meta-analysis was not applicable) |
|  | 13f | Describe any sensitivity analyses conducted to assess robustness of the synthesized results. | NO(meta-analysis was not applicable) |
| Reporting bias assessment | 14 | Describe any methods used to assess risk of bias due to missing results in a synthesis (arising from reporting biases). | Page 5, risk of bias assessment, and Table 2 |
| Certainty assessment | 15 | Describe any methods used to assess certainty (or confidence) in the body of evidence for an outcome. | Not applicable |
| **RESULTS** | | |  |
| Study selection | 16a | Describe the results of the search and selection process, from the number of records identified in the search to the number of studies included in the review, ideally using a flow diagram. | Figure 1 and page 7 (2.7. Search Summary) |
|  | 16b | Cite studies that might appear to meet the inclusion criteria, but which were excluded, and explain why they were excluded. | It was not applicable in this study. |
| Study characteristics | 17 | Cite each included study and present its characteristics. | Yes, in results section |
| Risk of bias in studies | 18 | Present assessments of risk of bias for each included study. | Yes, in results section, (3.1. Characteristics of Studies) |
| Results of individual studies | 19 | For all outcomes, present, for each study: (a) summary statistics for each group (where appropriate) and (b) an effect estimate and its precision (e.g. confidence/credible interval), ideally using structured tables or plots. | Table 2 and results sections: 3.3. Effects of MRT on Clinical Outcomes |
| Results of syntheses | 20a | For each synthesis, briefly summarise the characteristics and risk of bias among contributing studies. | Table 2 and pages 7, 8, and 9 |
|  | 20b | Present results of all statistical syntheses conducted. If meta-analysis was done, present for each the summary estimate and its precision (e.g. confidence/credible interval) and measures of statistical heterogeneity. If comparing groups, describe the direction of the effect. | Not apply (meta-analysis was not applicable) |
|  | 20c | Present results of all investigations of possible causes of heterogeneity among study results. | Not apply(meta-analysis was not applicable) |
|  | 20d | Present results of all sensitivity analyses conducted to assess the robustness of the synthesized results. | Not apply(meta-analysis was not applicable) |
| Reporting biases | 21 | Present assessments of risk of bias due to missing results (arising from reporting biases) for each synthesis assessed. | Not apply(meta-analysis was not applicable). Also, we had no missing of results |
| Certainty of evidence | 22 | Present assessments of certainty (or confidence) in the body of evidence for each outcome assessed. | Not apply(meta-analysis was not applicable) |
| **DISCUSSION** | | |  |
| Discussion | 23a | Provide a general interpretation of the results in the context of other evidence. | Yes, in discussion section |
|  | 23b | Discuss any limitations of the evidence included in the review. | Yes, page 13, Limitations and Suggestions |
|  | 23c | Discuss any limitations of the review processes used. | Yes, page 13, Limitations and Suggestions |
|  | 23d | Discuss implications of the results for practice, policy, and future research. | Yes, page 13, Limitations and Suggestions |
| **OTHER INFORMATION** | | |  |
| Registration and protocol | 24a | Provide registration information for the review, including register name and registration number, or state that the review was not registered. | CRD420251044596 |
|  | 24b | Indicate where the review protocol can be accessed, or state that a protocol was not prepared. | PROSPERO (CRD420251044596) |
|  | 24c | Describe and explain any amendments to information provided at registration or in the protocol. | PROSPERO (CRD420251044596) |
| Support | 25 | Describe sources of financial or non-financial support for the review, and the role of the funders or sponsors in the review. | Not applicable |
| Competing interests | 26 | Declare any competing interests of review authors. | Not applicable |
| Availability of data, code and other materials | 27 | Report which of the following are publicly available and where they can be found: template data collection forms; data extracted from included studies; data used for all analyses; analytic code; any other materials used in the review. | All data is available in the manuscript file and appendix |

*From:*  Page MJ, McKenzie JE, Bossuyt PM, Boutron I, Hoffmann TC, Mulrow CD, et al. The PRISMA 2020 statement: an updated guideline for reporting systematic reviews. BMJ 2021;372:n71. doi: 10.1136/bmj.n71

For more information, visit: <http://www.prisma-statement.org/>

**Supplemental Appendix 2: Search Strategy**

**Search Strategy for PubMed**

((((((Knee Osteoarthritis) OR (Osteoarthritis of the knee)) OR (Knee Osteoarthritides)) OR (Osteoarthritis of Knee)) OR (Osteoarthritis of Knees)) OR (Knee Arthritis)) AND ((((((((myofascial release) OR (muscle manipulation)) OR (myofascial therapy)) OR (fascial manipulation)) OR (trigger point therapy)) OR (deep tissue massage)) OR (active release technique)) OR (myofascial treatment))

**Search Strategy for Google Scholar**

(“Knee Osteoarthritis” OR “Osteoarthritis of the knee” OR “Knee Osteoarthritides” OR “Osteoarthritis of Knee” “Osteoarthritis of Knees” OR “Knee Arthritis”) AND (“myofascial release” OR “muscle manipulation” OR “myofascial therapy” OR “fascial manipulation” OR “trigger point therapy” OR “deep tissue massage” OR “active release technique” OR “myofascial treatment”)

**Search Strategy for Science Direct**

(“Knee Osteoarthritis” OR “Osteoarthritis of the knee” OR “Osteoarthritis of Knee” OR “Osteoarthritis of Knees”) AND (“myofascial release” OR “muscle manipulation” OR “myofascial therapy” OR “fascial manipulation” OR “trigger point therapy”)

**Search Strategy for Scopus**

(ALL(“Knee Osteoarthritis”) OR ALL(“Osteoarthritis of the knee”) OR ALL(“Knee Osteoarthritides”) OR ALL(“Osteoarthritis of Knee”) OR ALL(“Osteoarthritis of Knees”) OR ALL(“Knee Arthritis”)) AND (ALL(“myofascial release”) OR ALL(“muscle manipulation”) OR ALL(“myofascial therapy”) OR ALL(“fascial manipulation”) OR ALL(“trigger point therapy”) OR ALL(“deep tissue massage”) OR ALL(“active release technique”) OR ALL(“myofascial treatment”))

**Search Strategy for Web of science**

(((((ALL=(Knee Osteoarthritis)) OR ALL=(Osteoarthritis of the knee)) OR ALL=(Knee Osteoarthritides)) OR ALL=(Osteoarthritis of Knee)) OR ALL=(Osteoarthritis of Knees)) OR ALL=(Knee Arthritis) AND (((((((ALL=(myofascial release)) OR ALL=(muscle manipulation)) OR ALL=(myofascial therapy)) OR ALL=(fascial manipulation)) OR ALL=(trigger point therapy)) OR ALL=(deep tissue massage)) OR ALL=(active release technique)) OR ALL=(myofascial treatment)

**Search Strategy for ProQuest**

(“Knee Osteoarthritis” OR “Osteoarthritis of the knee” OR “Knee Osteoarthritides” OR “Osteoarthritis of Knee” “Osteoarthritis of Knees” OR “Knee Arthritis”) AND (“myofascial release” OR “muscle manipulation” OR “myofascial therapy” OR “fascial manipulation” OR “trigger point therapy” OR “deep tissue massage” OR “active release technique” OR “myofascial treatment”)
